# Immune Modulation and Stereotactic Radiation: Improving Local and Abscopal Responses

**DOI:** 10.1155/2013/658126

**Published:** 2013-11-14

**Authors:** Jing Zeng, Timothy J. Harris, Michael Lim, Charles G. Drake, Phuoc T. Tran

**Affiliations:** ^1^Department of Radiation Oncology, University of Washington, 1959 NE Pacific Street, P.O. Box 356043, Seattle, WA 98195, USA; ^2^Department of Radiation Oncology & Molecular Radiation Sciences, The Sidney Kimmel Comprehensive Cancer Center, Johns Hopkins Medicine, Baltimore, MD 21218, USA; ^3^Department of Oncology, The Sidney Kimmel Comprehensive Cancer Center, Johns Hopkins Medicine, Baltimore, MD 21218, USA; ^4^Department of Neurosurgery, Johns Hopkins Medicine, Baltimore, MD 21218, USA; ^5^Department of Urology, Johns Hopkins Medicine, Baltimore, MD 21218, USA

## Abstract

New and innovative treatment strategies for cancer patients in the fields of immunotherapy and radiotherapy are rapidly developing in parallel. Among the most promising preclinical treatment approaches is combining immunotherapy with radiotherapy where early data suggest synergistic effects in several tumor model systems. These studies demonstrate that radiation combined with immunotherapy can result in superior efficacy for local tumor control. More alluring is the emergence of data suggesting an equally profound systemic response also known as “abscopal” effects with the combination of radiation and certain immunotherapies. Studies addressing optimal radiation dose, fractionation, and modality to be used in combination with immunotherapy still require further exploration. However, recent anecdotal clinical reports combining stereotactic or hypofractionated radiation regimens with immunotherapy have resulted in dramatic sustained clinical responses, both local and abscopal. Technologic advances in clinical radiation therapy has made it possible to deliver hypofractionated regimens anywhere in the body using stereotactic radiation techniques, facilitating further clinical investigations. Thus, stereotactic radiation in combination with immunotherapy agents represents an exciting and potentially fruitful new space for improving cancer therapeutic responses.

## 1. Introduction 

Radiation therapy is generally considered a “local” treatment modality in cancer therapy, as radiation can only directly eradicate cancer cells inside the radiation field. However, more recent data has shown that the radiation response in the tumor and surrounding normal tissue activates a number of pathways that can help the host mount an immune response against tumors, especially in conjunction with immunotherapeutic agents. Here, we briefly summarize the interplay between cancers and the host immune system, the effects of radiation treatment on the immune system both locally and systemically, and the preclinical and clinical data on the combination of radiation treatment and immunotherapy. We also explore the existing data on the optimal dose and fractionation scheme of external beam radiation to be used with immunotherapy. 

## 2. Cancer and the Immune System

In order to understand how stereotactic radiation can enhance immunotherapy and lead to cancer control both locally and systemically, it is important to understand the interplay between the immune system and cancer. One proposed model by which the immune system responds to cancer has been termed “cancer immunoediting,” and it consists of three phases: elimination, equilibrium, and escape [1, 2]. Active research is underway to elucidate mechanisms involved in each phase of cancer immunoediting. The elimination phase is thought to involve both the innate and adaptive immune systems. When the immune system detects cells that are “non-self,” or similarly “altered-self” as is the case for cancer, cytokines can be released that activate the adaptive immune response to protect against foreign cells [3]. One such cytokine, IFN-*γ*, can be released by NK-cells to enhance macrophage cytotoxicity and dendritic cell maturation [4–6]. In turn, antigen-presenting cells can use the released tumor antigens to activate T-cells and the adaptive immune response [7]. Evidence of the elimination phase of cancer immunoediting can be inferred by the greater incidence of malignancies in humans and animals that are immunodeficient or immunosuppressed [2].

For the rare cancer cells that are able to survive the elimination phase, they can enter a state of equilibrium for variable periods of time and the tumor microenvironment in turn becomes permissive for tumor cell survival. Host immune cells such as lymphocytes and cytokines such as IL-12 and IFN-*γ* not only can suppress the cancer cells but also exert immense selective pressure for cells that are able to thrive in such an environment [8]. Antigen-presenting cell development is usually suppressed [9]. Cells typically express lower levels of costimulatory molecules (such as B7.1 and B7.2) and have high expression levels of coinhibitory molecules (such as B7-H1 also known as PD-L1) (for a more comprehensive review of immune stimulatory and inhibitory checkpoints [10]). 

To fully escape the immune system cancer cells typically either (1) mutate to decrease expression of antigens that currently induce an immune response, (2) lose expression of MHC class I proteins so antigens are no longer presented to the immune system, or (3) have an aberrant antigen processing pathway so antigens cannot be loaded onto the MHC class I molecules [2]. All these preceding complex interplay factors ultimately lead to cancer cell escape. Although immune escape is required for clinically relevant tumor growth and development, the immune system is still active, and manipulations through immunotherapy and radiation can potentially result in lasting tumor directed responses and cancer control.

## 3. Radiation and the Immune System

Radiation is a long-standing treatment modality for cancer therapy, but it has historically been considered to be immunosuppressive. Decades ago, it was noted that following exposure to low dose total body irradiation, death was typically due to bone marrow failure. In fact, total body irradiation is commonly in use today as part of the conditioning/preparatory regimen prior to bone marrow transplantation [11]. Even localized radiation therapy, such as radiation to the chest or central nervous system, can cause lymphopenia [12, 13]. This is thought to be mainly due to the irradiation of the circulating blood pool and the inherent radiation sensitivity of immune cells to even low doses of radiation (<1 Gy) [13, 14]. Although radiation is predominately considered a “local” treatment modality in that radiation only has direct effects on tumor cells in the field of irradiation, it is clear that the effect of radiation on the immune system has implications for the entire host.

Even though radiation has long been thought to suppress the immune system, abundant evidence also exists that in certain settings radiation can also enhance the activity of the immune system against cancer. Established tumor cells escape the immune system by frequently losing presentation of antigens through various genetic and epigenetic mechanisms. One way to reestablish presentation of tumor antigens is through the release of tumor antigens upon cancer cell death. The direct cytotoxic effects of radiation can lead to the release of tumor specific antigens, which can then direct antigen-presenting cells to induce a T-cell immune response [15]. However, not all modes of cancer cell death can induce an immune response. Although dendritic cells can present tumor antigens to T-cells, successful activation of tumor antigen-specific T-cell immunity involves further “danger” signals to enhance T-cell activation. One mechanism of achieving this enhanced T-cell activation following tumor irradiation is via the secretion of the HMGB1 protein by dying irradiated tumor cells and binding of HMGB1 on TLR4 expressed by dendritic cells. Irradiated tumor cells can also release other “danger” signals such as heat shock proteins (HSPs) [16]. In addition, radiation-induced cell death (and some other cell killing modalities) is associated with translocation of calreticulin to the cell surface, which facilitates phagocytosis of tumor cells by dendritic cells [17]. Therefore, during radiation-induced cell death, both tumor antigen release and presentation are enhanced, helping to activate an immune response [18]. These specific events following radiation-induced tumor cell killing have led to the concept of utilizing radiation treatment as a method of “*in situ* vaccination” [19, 20].

Besides decreasing the level of tumor specific antigen expression, another method of immune escape often employed by tumor cells is the decreased expression of MHC molecules. Even relatively low doses of radiation that do not induce cell kill can still cause an increase in tumor cell expression of MHC class I molecules [21]. MHC class I molecules present endogenous peptides to cytotoxic T-cells through peptide-MHC complexes. After radiation, there is an increased pool of peptides for antigen presentation due to faster degradation of existing proteins and enhanced translation of proteins. In addition, new proteins are made in response to radiation, resulting in new peptides presented by MHC class I molecules [21]. This all enhances the ability of tumor cells to trigger a tumor specific T-cell response. 

Radiation can also increase tumor cell susceptibility to immune mediated cell kill by altering cellular surface expression of a number of molecules. Even at sublethal doses, radiation increases Fas gene expression in tumor cells, which leads to increased susceptibility to cytotoxic T-cell mediated killing [22, 23]. Other molecules that help mediate cytotoxic T-cell killing such as ICAM-1 are also increased after irradiation. These findings have been confirmed in multiple human cancer cell lines, and it has been demonstrated *in vitro* that cells exposed to nonlethal levels of radiation are more susceptible to cytotoxic T-cell killing than nonirradiated cells [22].

Another mechanism by which radiation can enhance tumor-specific immune response is through direct elimination of part of the tumor population or immune cell population that is detrimental to mounting an effective immune response. Radiation treatments can drastically decrease the tumor cell population and thus in turn decrease immune tolerance stemming from chronic presence of a large amount of tumor antigen [10]. Moreover, established tumors are composed of generally more immunoresistant clones that have been forged from selective pressure during immunoediting. Radiation-induced cancer cell killing does not distinguish between cells more susceptible to the immune system versus cells that are more resistant. Thus, radiation treatment may eliminate more immunoresistant tumor clones, allowing for more effective immune mediated killing of the remaining tumor clonogens. All of these mechanisms described above for enhancing the immune system activity against tumors act locally against tumors being irradiated, but a local immune response can also translate into systemic immunity, inducing “abscopal” effects at tumors distant to the irradiated site [24].

### 3.1. Preclinical Data

The combination of radiation and immunotherapy has been tested in preclinical animal models confirming the potential synergy between the two modalities. We will next highlight some select studies with a preference towards those exploring stereotactic or hypofractionated radiation. In a mouse orthotropic cell line glioma model, stereotactic radiosurgery (SRS) with 10 Gy was tested in combination with anti-PD-1 antibodies. Binding of PD-1 to its ligands B7-H1 (PD-L1) or B7-DC (PD-L2) induces apoptosis or exhaustion of activated immune cells, and blocking this inhibitory immune checkpoint has been shown to enhance antitumor activity. Combined modality treatment with SRS and anti-PD-1 antibodies led to long term cures in a subset of mice, which was not seen with either treatment modality alone [25]. Post treatment analysis of brain tissue showed increased cytotoxic T-cells in the combined modality arm and decreased regulatory T-cells. Beyond local tumor control in the brain, there was also evidence of abscopal or systemic antitumor effect. When mice cured of experimental brain tumors were rechallenged months later in the flank with the isogenic tumor cells, they failed to establish flank tumors, unlike naïve mice that had not previously seen the tumor cell line.

Using breast cancer mouse models, different radiation fractionation schemes (20 Gy × 1, 8 Gy × 3, or 6 Gy × 5 fractions in consecutive days) were tested in combination with the immune checkpoint inhibitor, anti-CTLA-4 antibody, by examining two tumor sites, a primary site that was irradiated and a secondary site that was unirradiated [26]. Radiation alone was able to deter tumor growth at the primary site but had no effect on the secondary site, while antibody therapy alone had no effect on either tumors. However, when the radiation and antibody was combined, there was enhanced tumor response at the primary site and an abscopal effect at the secondary site. Interestingly, the abscopal effect observed at the secondary tumor sites was only in mice treated with the combination of CTLA-4 blockade and fractionated radiotherapy and not the single hypofractionated dose. Using similar mouse models of breast cancer, the abscopal effect resulting from combining radiation and CTLA-4 blockade was abrogated with depletion of cytotoxic T-cells, demonstrating the dependency of the abscopal effect on the immune system [27]. A more recent study examined the effect of combination therapy with multiple immunostimulatory and immunoinhibitory monoclonal antibodies with radiation using mouse models of triple-negative breast cancer [28]. The effects on established triple-negative primary tumors using single hypofractionated dose and fractionated radiation with immunotherapy were similarly impressive. Similar to the previous studies, functionally active tumor-reactive lymphocytes persisted within irradiated tumors, but abscopal responses were not tested in these models.

Beyond using agents to target receptors that downregulate T-cell activity such as CTLA-4 and PD-1, other studies have also looked at combining radiation treatment with cytotoxic T-cell mediated adoptive immunotherapy [23]. Mice injected with CEA+ tumor cells subcutaneously were irradiated with 8 Gy in single fraction and given CEA-specific cytotoxic T-cells. When either radiation or CEA-specific cytotoxic T-cells were administered alone, there was no substantial decrease in tumor growth, although radiation induced an initial tumor regression before tumor regrowth. However, combination of the two treatments resulted in drastic and durable tumor response. Approximately half of treated mice experience complete resolution of tumor. Another study tested the combination of tumor irradiation with IL-2 therapy in an animal model looking at lung metastasis from renal adenocarcinoma [29]. Mice received radiation to the left lung, followed by systemic IL-2 therapy, which resulted in tumor reduction in both lungs. There appears to be radiation dose dependence, as increasing doses of radiation resulted in not only improved tumor control in the irradiated site but also improved tumor control outside the radiation field. Additionally, the timing and sequencing of immunotherapy and radiotherapy were proved to be critical, as demonstrated in a mouse model of prostate cancer treated with a poxvirus-based tumor vaccine [30].

### 3.2. Clinical Data

Early phase clinical data supports the use of radiation therapy in combination with immunotherapy. A randomized phase II trial tested whether radiation in combination with a poxviral vaccine encoding prostate-specific antigen (PSA) can induce a PSA-specific T-cell response, compared with radiation alone [31]. The radiation dose and fractionation were standard for definitive treatment of prostate cancer at the time of the trial, typically at least 70 Gy in 1.8–2.0 Gy daily fractions given over 7-8 weeks. The priming vaccine consisted of recombinant vaccinia expressing PSA, costimulatory molecule B7-1, followed by monthly booster vaccines with recombinant PSA. Local GM-CSF and low dose systemic IL-2 were given with the vaccines. Patients received standard external beam radiation therapy between the fourth and sixth vaccinations. Combination of radiation plus vaccine caused an increase in PSA-specific T-cells of at least 3-fold versus no detectable increases in the radiotherapy-only arm (*P* < 0.0005). There was also “epitope spreading”—generation of T-cells specific to known prostate-associated antigens not found in the vaccine—providing indirect evidence of immune mediated tumor killing, supporting the *in vitro* evidence that radiation cell kill can lead to an increased peptide pool for antigen presentation [31]. Another phase I clinical trial tested direct injection of autologous immature dendritic cells (DCs) into tumor under radiotherapy in advanced hepatoma patients. Radiation was delivered in a single fraction of conformal radiotherapy to 8 Gy. Of 14 patients entered on the trial, there were two clinically evident partial responses and four minor responses. For 10 patients who underwent immunologic response evaluation after vaccination, an AFP-specific immune response was seen in at least 7 patients, and 6 patients showed an increased NK cell cytotoxic activity [32]. This provides further evidence that radiation and immunotherapy can lead to immune mediated cell kill in patients with established tumors who have already undergone other cancer treatment modalities.

In addition to obtaining local control of established tumors, radiation treatment and immunotherapy also show promise in the metastatic cancer setting. A phase I/II study looked at combining local radiation to enhance tumor immunogenicity with direct intratumoral injection TLR9 agonist [33]. Out of 15 patients with mycosis fungoides, 5 clinically meaning responses were seen at distant, untreated sites. The treated sites showed a significant reduction of CD25^+^Foxp3^+^ T-cells that could be either cancer cells or regulatory T-cells. Recently, multiple separate reports of patients with metastatic melanoma or renal cell carcinoma have demonstrated abscopal effects after treatment with radiation and immune modulatory therapies. A phase I study treated metastatic melanoma or renal cell carcinoma patients with one, two, or three doses of stereotactic body radiation (SBRT) (20 Gy per fraction) with the last dose administered 3 days before starting IL-2 [34]. Among 12 patients, eight (or 66.6%) achieved a complete (CR) or partial response (PR) with abscopal responses observed. One case report presented a metastatic melanoma patient who had progressive disease on ipilimumab, an anti-CTLA-4 antibody, who received 28.5 Gy in three fractions to a paraspinal mass [35]. There was initially no response for one month following the radiation. However, when an additional dose of ipilimumab was given one month later, there was subsequent regression in the irradiated tumor, as well as other tumors in the patient that were not irradiated. Similarly, there is a more recent case report of a metastatic melanoma patient treated with ipilimumab and stereotactic radiation (54 Gy in 3 fractions). Patient only received treatment to two of seven metastatic liver lesions, but developed complete resolution of cancer all over the body, and is free of melanoma one year after the radiation treatment [36]. Finally, a phase I/II study in patients with metastatic castration-resistant prostate cancer explored ipilimumab treatment, alone or with radiation therapy [37]. The radiation was 8 Gy in a single fraction to metastatic lesions. In the highest dose group at 10 mg/kg, out of 50 patients, 8 patients saw PSA declines of at least 50%, 1 patient had complete response, and 6 patients had stable disease. The relative efficacy of ipilimumab alone or with radiation is being tested in phase III clinical trials.

## 4. Radiation Treatment

### 4.1. Treatment Delivery Methods

The optimal radiation treatment in conjunction with immunotherapy is unknown and may vary depending on the specific type of immunotherapy agent. Radiation can be delivered through a number of different methods. Most radiation treatment is delivered using external beam radiation, where radiation is generated outside of the patient body, and the radiation beam is directed to the target area in the patient based on image guidance, such as a planar X-ray or a CT scan. This is by far the most readily available and commonly delivered form of radiation. The radiation beam is either generated by a radioactive source (such as Cobalt-60) or a machine such as a linear accelerator (for photon radiation) or a cyclotron (for proton and neutron radiation). 

Other methods of radiation delivery include brachytherapy, where a radioactive source is inserted inside or adjacent to the site to be treated. The most widely known example for this is low dose rate (LDR) prostate cancer brachytherapy, where radioactive seeds are permanently implanted inside the prostate. The radioactivity decays over time and delivers the prescribed dose of radiation. Another common brachytherapy scenario is endobronchial brachytherapy, where a tumor inside the airway can be treated with a radioactive source that is placed next to the tumor via a hollow catheter and is removed after treatment is complete. Endometrial cancer and cervical cancer are also often treated with brachytherapy, where radioactive sources are inserted next to the tumor for a calculated length of time and then removed. 

There are also unsealed source radiation therapies, such as radionuclides conjugated to monoclonal antibodies, that can target tumor antigens specifically. These unsealed sources are often delivered intravenously and travel to their intended tumor cells. Examples of this include ibritumomab tiuxetan (anti-CD20 monoclonal antibody conjugated to a molecule that chelates Yttrium-90) and tositumomab (anti-CD20 monoclonal antibody linked to Iodine-131). Besides tumor targeting with monoclonal antibodies, some radionuclides have biological properties that preferentially localize them to a specific organ, such as iodine to thyroid, or samarium and strontium to bone. Radioactive isotopes of iodine are used to treat thyroid cancer, and strontium and samarium can be used to treat cancer that has spread to the bone.

### 4.2. Radiation Dose

Studies on the relationship between radiation and immune response have generally found a dose dependency. In an *in vitro* study looking at the effect of irradiation on antigen presentation by MHC class I molecules, cell surface expression of MHC class I molecules was increased for many days in a radiation dose-dependent manner [21]. In an animal model looking to combine radiation with IL-2 in metastatic renal adenocarcinoma to the lung, authors also saw that higher radiation dose resulted in greater tumor reduction, both inside the radiation field, and in the contralateral unirradiated lung [29]. In this study, 8 Gy caused a more pronounced increase in H-2Kd class I MHC antigen than 3 Gy. 

### 4.3. Radiation Fractionation

In addition to dose, the issue of fractionation has also been explored. In one study, mice bearing B16-OVA murine melanoma were irradiated up to 15 Gy, given in various size fractions [38]. For single fractions, tumor control and number of tumor-specific T-cells were radiation dose dependent. However, at the highest dose, there was also an increase in regulatory T-cells, which tend to downregulate the immune response. Fractionated irradiation at 7.5 Gy/fraction seemed to produce the best tumor control and tumor specific T-cell response while still maintaining low regulatory T-cell numbers. Another study also supports fractionating radiation treatment in conjunction with immunotherapy, by testing anti-CTLA-4 antibodies with radiation in a mouse breast cancer model. Mice were treated with 20 Gy × 1, 8 Gy × 3, or 6 Gy × 5 fractions in combination with 9H10 monoclonal antibody against CTLA-4. Authors found that fractionated but not single-dose radiotherapy induces an abscopal effect when used with anti-CTLA-4 antibody [26]. However, there may be a limit to fractionation, as reported in a preclinical study comparing ablative radiation doses against fractionated radiation [39]. Ablative radiation, such as a single dose of 20–25 Gy, dramatically increased T-cell activity and tumor control. When 5 Gy × 4 fractions given over 2 weeks were compared against a single 20 Gy dose, radiation-initiated immune responses and tumor reduction appeared to be abrogated by the fractionated radiation (for a more in-depth review on dose and fractionation with immune modulatory drugs [40]).

### 4.4. Radiation Treatment Technology Advances

**Figure 1:**
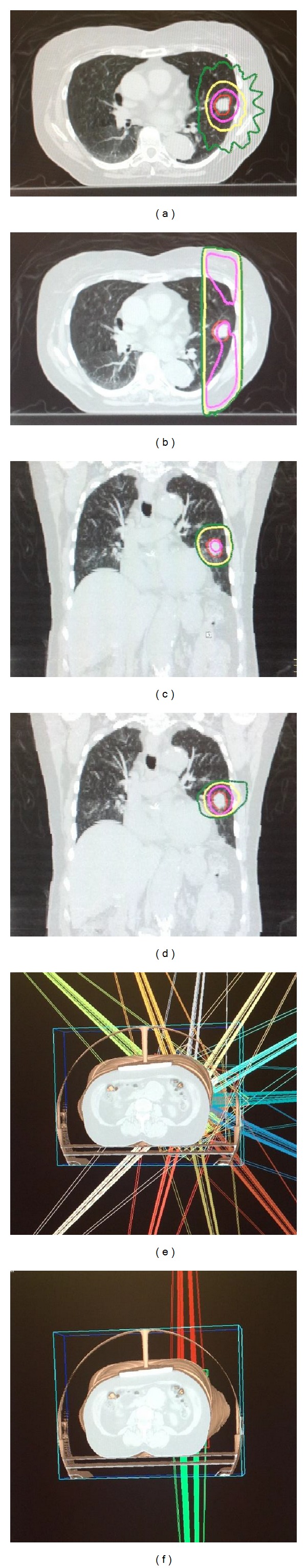
Stereotactic radiosurgery versus conventional radiation. Left three images depict stereotactic radiosurgery for a lung tumor: (a) shows a patient's transverse CT image through the lung tumor (red = tumor outline, purple = everything inside the line is receiving at least 100% of the full prescribed radiation dose, yellow = everything inside the line is receiving at least 60% of the full prescribed radiation dose, and green = everything inside the line is receiving at least 30% of the full prescribed radiation dose); (c) shows the same patient in coronal CT view; and (e) shows the 11 beams of radiation centered on the tumor, to generate a radiation plan that is highly focused on the tumor. Right three images depict a more traditional radiation plan for a lung tumor (colored lines defined same as images on the left): (b) shows a patient's transverse CT image through the lung tumor; (d) shows the same patient in coronal CT view; and (f) shows the 2 beams of radiation centered on the tumor. In comparison to conventional radiation, stereotactic radiosurgery is able to focus the high and medium radiation dose regions around the tumor while sparing normal tissues and organs.

The possibility of single or extreme hypofractionation (very few radiation treatment fractions, such as 3–5 fractions) with high dose radiation in combination with immunotherapy has become a reality in the clinic by way of recent technologic advances that have made it possible to safely deliver stereotactic ablative body radiation (SABR) to sites throughout the body (Figure 1). Stereotactic radiation/radiosurgery involves highly conformal and targeted treatment [41]. Stereotactic radiosurgery has been in use for several decades in treating brain tumors using a rigid frame fixed to the head to allow precise spatial positioning of target lesions. In order to safely deliver a large dose of radiation to normal organs, multiple beams from different angles are utilized, centered on the tumor. The incorporation of 3-dimensional anatomical information from CT scans and more robust computational power has made it possible to generate more sophisticated radiation treatments that may use more than a dozen radiation beams, which was not feasible when radiation planning was based on planar X-ray anatomy. Historically, radiosurgery has been most commonly used for lesions in the brain, because the brain is a fixed structure relative to the calvarium that is amenable to rigid fixation with a frame-based system which allows for precise target localization. However, more reproducible patient immobilization coupled with image-guided radiation treatment machines, which have on-board ability to perform a variety of imaging prior, during and/or after radiation delivery, has made it possible to treat any site in the body, such as the lungs, prostate, liver, and pancreas. In addition, motion tracking and management technology now are in clinical use, so that respiratory motion and other internal organ motions can be accounted for in radiation planning and delivery. Much clinical experience has proven the safety and efficacy of stereotactic radiation in multiple body sites.

### 4.5. Particle Radiation Therapy

While most stereotactic radiation treatments are delivered with photon external beam radiation, there is increasing availability of particle therapy, particularly proton therapy. Particles such as protons, neutrons, and carbon ions have different physical characteristics than photon radiation, which impart dramatically different radiation dose depositions in biological matter [42]. For photon radiation, the radiation beam enters the patient's body, deposits energy, and causes damage along its pathway, and a substantial amount of the radiation beam exits in the patient's body on the other side. Charged particles, such as protons and carbon ions, enter the patient's body and only travel for a limited range before losing energy and depositing the full radiation dose. There is little to no exit dose on the other side. 

**Figure 2:**
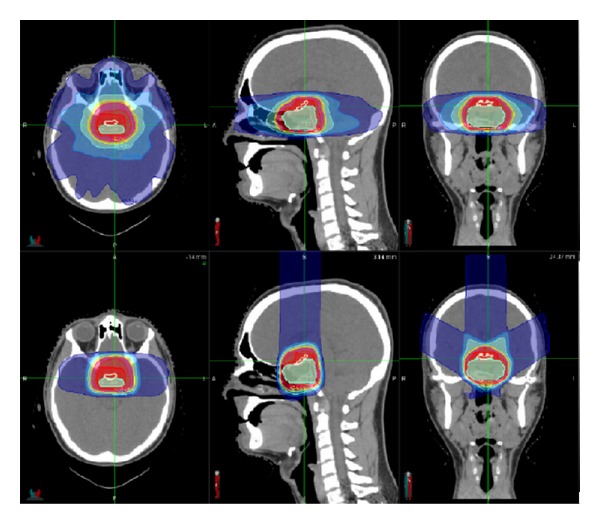
Proton radiation dose distribution. Patient with a tumor in the pituitary gland, receiving proton radiation to the tumor (innermost cyan shaded area). Upper three images show the radiation dose distribution from a photon radiation treatment plan using multiple converging beams on axial CT image (left upper), sagittal CT image (middle upper), and coronal CT image (right upper). Lower three images show the radiation dose distribution from a proton radiation treatment plan using three proton beams on axial CT image (left lower), sagittal CT image (middle lower), and coronal CT image (right lower). For all images, innermost red line = everything inside the line is receiving at least 100% of the full prescribed radiation dose, and outermost blue line = everything inside the line is receiving at least 20% of the full prescribed radiation dose. Note that the high radiation dose region (inside the red line) looks similar between the photon and proton plan, but the lower radiation dose region (inside the blue line) is much smaller with proton radiation than photon radiation.

This difference in the physical properties of a photon radiation beam versus a proton radiation beam can lead to drastically different radiation dose distributions inside the patient's body. As described above, stereotactic photon radiosurgery has the ability to use many converging radiation beams to form a high dose region precisely around the tumor. However, the use of many converging beams also spreads out a low dose of radiation to a larger area. With charged particle radiation, such as proton radiation, very few beams are typically utilized, so there is not a large low dose region around the tumor, as illustrated in Figure 2. Since hematopoietic cells are extremely sensitive to radiation, it is unknown yet whether this would have an impact on the interplay between radiation and immunotherapy. 

Besides the physical properties, particle radiation therapy can also have different biological effects than photon radiation. It is well known that different types of radiation particles have different relative biological effectiveness (RBE). For the same amount of energy deposited into tissue, RBE allows for the comparison of biological effectiveness of different types of radiation. Radiation particles with higher RBE values can be more damaged by energy deposition than particles with lower RBE values. Photon radiation in routine clinical use typically has RBE of 1. Charged particles such as protons have RBE that can vary along the beam pathway, due to the fact that most of the energy deposition from charged particle beams happens near the end of the beam. A commonly used RBE for proton radiation is around 1.1, whereas neutron radiation has RBE around 2–5. It is unknown whether this varying range of RBE values has differing effects on the immune system.

Given the wide range of radiation treatment methods and modalities available today, the biological effects of each of these methods on the immune system will need further exploration in order to optimize the combination between immunotherapy and radiation therapy.

## 5. Conclusion

There is increasing evidence that radiation can work to produce quantitatively superior results with immunotherapy for local tumor control at the site of irradiation, but much excitement is focused on a completely different qualitative responses than can emerge between radiation and immunotherapy, systemic abscopal cancer eradication. While the optimal radiation dose, fractionation, and modality to be used in combination with immunotherapy remain to be determined, some preclinical evidence suggests that higher doses of radiation such as those delivered during hypofractionated regimens generate more immunologic responses. Recent technologic advances in clinical radiation therapy have now made it possible to deliver ablative radiation doses precisely to tumors using stereotactic radiation techniques such as SBRT/SABR. Thus, stereotactic radiation in combination with immunotherapy agents represents an exciting and potentially fruitful new space for improving cancer therapeutic responses.
